# Retraction: Effects of Dietary Fiber Interventions on Glycemic Control and Weight Management in Diabetes: A Systematic Review of Randomized Controlled Trials

**DOI:** 10.7759/cureus.r175

**Published:** 2025-04-01

**Authors:** Syed Ali Hussein Abdi, Syed Imran Ali Abdi, Mohamed H Ali, Noor A Balani, Nazik A Balani, Hestia L Jacob, Ariana Seyfi, Gadeer H Al Shabout, Dena N Hamza, Alan Deiar Al-Talabani, Ramadan Khan

**Affiliations:** 1 Internal Medicine, Ajman University, Ajman, ARE; 2 Medicine, Gulf Medical University, Ajman, ARE; 3 Medicine, Thumbay University Hospital, Ajman, ARE; 4 Surgery, Ajman University, Ajman, ARE; 5 Medicine and Surgery, Ajman University, Ajman, ARE; 6 Internal Medicine, D.G Khan Medical College, Dera Ghazi Khan, PAK

The Editors-in-Chief have retracted this article. An investigation by the journal found evidence of authorship manipulation. The Editors-in-Chief therefore no longer have confidence in the provenance and reliability of the article contents. The authors agree with the decision to retract.

